# High-speed and high-sensitivity multi-gas detection based on parallel heterodyne LITES sensor

**DOI:** 10.1038/s41377-026-02385-4

**Published:** 2026-06-29

**Authors:** Haiyue Sun, Shunda Qiao, Ying He, Yuanzhi Wang, Jinfeng Hou, Chu Zhang, Yongkang Dong, Yufei Ma

**Affiliations:** 1 National Key Laboratory of Laser Spatial Information, Harbin Institute of Technology, Harbin, 150000 China; 2 Zhengzhou Advanced Research Institute of Harbin Institute of Technology, Zhengzhou, 450000 China

**Keywords:** Optical spectroscopy, Optical metrology

## Abstract

A parallel heterodyne light-induced thermoelastic spectroscopy (PH-LITES) sensor is proposed for high-speed and high-sensitivity multi-gas detection for the first time. Within the collaborative signal enhancement architecture (CSEA), high sensitivity and high-speed detection are achieved at the physical layer. A self-designed cylindrical multi-pass cell (MPC) with a recorded high optical path length to volume ratio (OPL/V = 37.4 cm^–2^) and a four‑tine quartz tuning fork (QTF) with a low resonant frequency (*f*_0_ = ~7.9 kHz) work synergistically to enhance detection responsivity, establishing a robust foundation for highly sensitive detection of low-concentration gas mixtures. High‑speed capability is enabled by parallel heterodyne modulation, where a single QTF is excited to generate a composite transient response signal, allowing for the rapid, simultaneous acquisition of spectral information from multiple gases. At the information layer, intelligent processing is implemented via a collaborative intelligent processing architecture (CIPA) integrating convolutional neural networks (CNN), a hybrid attention mechanism (HAM), and bidirectional long short-term memory (BiLSTM). The CNN-HAM-BiLSTM architecture performs feature extraction, attention-based enhancement, and temporal modeling to enable accurate concentration retrieval from the parallel spectra derived from a single QTF output. Experimental validation using methane (CH_4_) and acetylene (C_2_H_2_) achieved minimum detection limits (MDLs) of 378 ppb and 285 ppb, respectively, within a 4 s scanning time. The proposed system offers an efficient solution for applications requiring rapid and sensitive multi-gas detection.

## Introduction

In recent years, rapid, sensitive, and simultaneous multi-gas detection has become essential in several cutting-edge strategic fields. For example, in origin-of-life studies around deep-sea vents, it is crucial to track subtle variations in mantle-derived trace gases such as methane (CH_4_) and hydrogen sulfide (H_2_S), along with their coupling to microbial activity^1,2^. In environmental monitoring, the rapid identification and differentiation of complex chemical threats, including release of nerve agents and toxic industrial chemicals, are paramount for mitigating risks^3,4^. Similarly, in planetary exploration, the detection of gases like CH_4_ and acetylene (C_2_H_2_) may indicate geological or biological activity^5–7^. Traditional single-gas sensors cannot fully meet the demands of these application scenarios. Thus, high-speed and high-sensitivity multi-gas detection is instrumental for advancing these and other strategic domains.

Recent advances in laser spectroscopy have been widely reported^8–12^, and laser absorption spectroscopy (LAS) is widely used for trace gas sensing due to its high sensitivity and rapid response^13–23^. Among LAS techniques, quartz-enhanced photoacoustic spectroscopy (QEPAS), first introduced in 2002, replaces the traditional microphone in photoacoustic spectroscopy (PAS) with a quartz tuning fork (QTF)^24^. The QTF offers high quality factor, small size, and low cost, making QEPAS a prominent research topic^25–32^. Heterodyne QEPAS (H-QEPAS) was demonstrated in 2017, incorporating heterodyne modulation to rapidly calibrate the QTF’s resonant frequency (*f*_0_)^33^. However, as a contact-based detection method, QEPAS is susceptible to corrosion from reactive target gases such as H₂S and hydrogen chloride (HCl). To overcome this, another QTF-based approach, light-induced thermoelastic spectroscopy (LITES), emerged in 2018^34^. LITES decodes gas information by measuring the thermoelectric signal generated on the QTF when laser light passes through the gas sample^35–42^. This non-contact approach shows great potential across various applications.

Currently, LITES suitable for rapid multi-gas detection are scarce. Existing multi-gas laser absorption methods typically adopt time-division multiplexing (TDM), spatial-division multiplexing (SDM), or frequency-division multiplexing (FDM), each with inherent limitations^43–48^. TDM is constrained by time-sharing limitations, while FDM requires lasers with distinct modulation frequencies, making it incompatible with QTF of fixed *f*₀. SDM configurations often incorporate multi-pass cells (MPCs) with multiple optical channels, multiple QTFs, and associated electronics, resulting in complex and bulky systems. These limitations collectively hinder the application of existing quartz-based techniques for fast multi-gas detection. Beyond quartz-based techniques, several other laser absorption spectroscopy methods have also been developed for rapid simultaneous multi-gas detection^49–52^. To the best of our knowledge, no prior study has reported fast and highly sensitive multi-gas detection based on LITES sensor.

To overcome these limitations, we propose for the first time a parallel heterodyne LITES (PH‑LITES) sensor that synergistically integrates collaborative signal enhancement architecture (CSEA) at the physical layer with collaborative intelligent processing architecture (CIPA) at the information layer. High sensitivity and high-speed detection are achieved within the CSEA. For high sensitivity, self-designed cylindrical MPC with a recorded high optical path length to volume ratio (OPL/V = 37.4 cm^–2^) is combined with a four‑tine QTF with a low resonant frequency (*f*_0_ = ~7.9 kHz). For high-speed detection, parallel heterodyne modulation is implemented, in which a single QTF is excited to generate a composite transient response signal, allowing simultaneous spectral acquisition of multiple gases within seconds. Multi‑component capability is accomplished by the CIPA, which incorporates feature extraction, hybrid attention mechanisms, and temporal modeling to disentangle overlapping spectral features from the composite signal, thereby enabling accurate concentration retrieval of mixed gases from a single QTF output. To the best of our knowledge, this work represents the first demonstration of a LITES‑based sensor capable of rapid, sensitive, and simultaneous multi‑gas detection.

## Results

### Overall design of the PH-LITES sensor

**Fig. 1:**
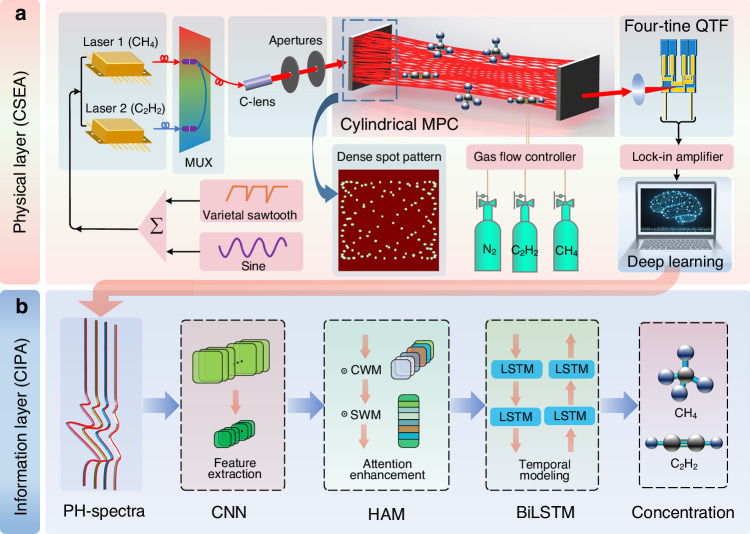
The overall architecture of the PH-LITES sensor. **a** Physical layer. **b** Information layer

The overall architecture of the PH-LITES sensor, which comprises physical and information layers, is illustrated in Fig. 1.

At the physical layer, the CSEA integrates a self-designed, absorption-enhanced cylindrical MPC and a detection-enhanced four-tine QTF, both of which surpass the performance levels reported in existing researches. This configuration ensures the purity of the original training signals and effectively suppresses noise interference. The sensor’s performance is experimentally validated using CH_4_ and C_2_H_2_ as representative gases. A varietal wave, combined with a high-frequency sine wave, was applied to the lasers for CH_4_ (1.65 μm) and C_2_H_2_ (1.53 μm). The lasers were coupled via a wavelength division multiplexer (MUX) and co-propagated along a shared optical path within the cylindrical MPC. This design leverages an ultra-dense spot-overlapping pattern to amplify the absorption signals from the multi-component gases. After undergoing hundreds of reflections, the light is incident on the four-tine QTF, where it generates a rapidly varying, mixed optical heat source localized on the QTF surface. This heat source, in turn, excites a corresponding transient parallel signal, enabling fast response to CH_4_ and C_2_H_2_. The resulting signal is demodulated by a lock-in amplifier and then fed into the information layer for model training, learning, and concentration inversion.

At the information layer, the CIPA is presented for inversion prediction in the PH-LITES sensor. This architecture integrates convolutional neural networks (CNN), hybrid attention mechanism (HAM), and bidirectional long short-term memory (BiLSTM) to address the core challenges in multi-component gas detection via parallel heterodyne spectroscopy, such as feature overlap, background noise, and insufficient utilization of spectral-temporal correlations. Before the preprocessing stage, a moving average smoothing filter is applied to suppress high-frequency noise. The CIPA adheres to the workflow of “feature extraction, attention enhancement, temporal modeling” to ultimately realize synchronous quantitative analysis of multiple gases, thereby facilitating the simultaneous and rapid quantification of CH_4_ and C_2_H_2_ in gas mixtures.

### CSEA design at the physical layer

The efficacy of deep learning networks in achieving accurate data prediction is critically dependent on the quality of the acquired signals. Consequently, the CSEA at physical layer demands a high signal-to-noise ratio (SNR) to avoid noise masking effective signals or contaminating the original training dataset. Enhancing the SNR presents two primary challenges: weak absorption signals arising from low gas concentrations, and the demand for detectors with high responsivity to optical signals.

**Fig. 2:**
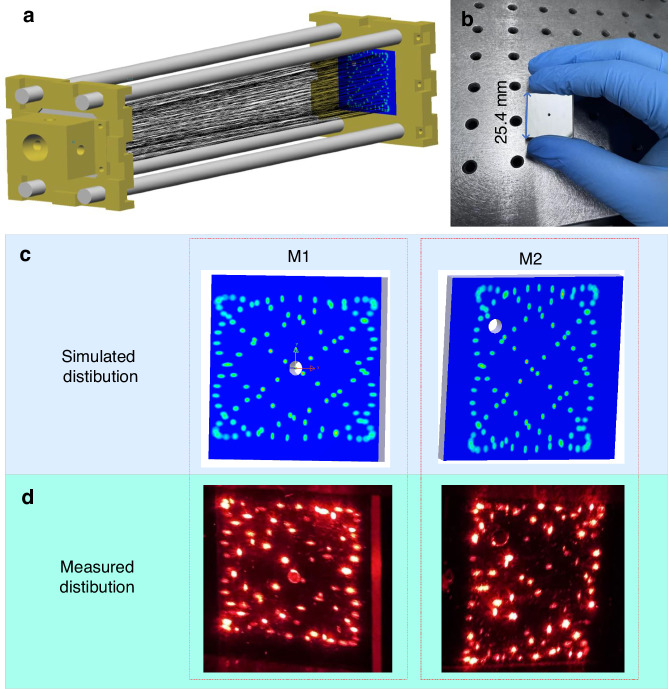
The novel cylindrical MPC design with ultra-dense spot pattern. **a** Simulated model of the cylindrical MPC. **b** Real picture of the cylindrical mirror. **c** Simulated distribution. **d** Measured distribution

To overcome the first challenge, we refer to the Beer-Lambert law, which indicates that absorption signal strength can be enhanced by increasing the optical path length (OPL). Spherical-mirror-based MPCs are widely used to extend the OPL, but face a trade-off between long OPL and compact volume (V). This study circumvents the limitations of traditional spherical MPCs by developing a cylindrical MPC with an ultra-high OPL/V ratio. The MPC consists of two orthogonally arranged cylindrical mirrors (M1 and M2) with different curvatures. This configuration leverages the structural asymmetry along the x- and y-axes to introduce astigmatism, thereby generating a dense laser spot pattern. With both the incident and exit apertures initially centered on M1, the formulas for the spot positions on the two axes can be simplified as follows:

1 $${x}_{2n}=A\,\sin (2n{\theta }_{x})$$

2 $${y}_{2n}=B\,\sin (2n{\theta }_{y})$$

where *A* and *B* represent the maximum position coordinates of the spot on the two axes, respectively. The variables *θ*_x_ and *θ*_y_ denote the angular step of the spot displacement on the respective axes between consecutive reflections on the same mirror, given by:

3 $$\cos (2{\theta }_{x})=1-\frac{d}{{f}_{x}}$$

4 $$\cos (2{\theta }_{y})=1-\frac{d}{{f}_{y}}$$

where *f*_x_ and *f*_y_ are the focal lengths of the two cylindrical mirrors, respectively, and *d* is the distance between them. Different values of *θ*_x_ and *θ*_y_ correspond to different spot patterns. The light exits the MPC when the reflected beam returns to the origin point on M1. With parameters set to *d* = 159 mm, *f*_x_ = 360 mm, and *f*_y_ = 591 mm, the calculated ratio is *θ*_x_:*θ*_y_ = 3:4, which is consistent with the dense swirling spot pattern featuring same frequency ratio. Calculated reflection times (*γ*) is 241. The ray tracing model is shown in Fig. 2.

To facilitate the rational arrangement of other experimental devices, this study alternatively positions the exit aperture on M2. The mirrors have a side length of 25.4 mm, resulting in actual parameters of *γ* = 240, OPL = 38.3 m, V = 102.6 mL, and an OPL/V ratio of 37.4 cm^–2^. The spots are uniformly and densely distributed on the mirror surface, improving mirror utilization. The OPL was calibrated using tunable diode laser absorption spectroscopy (TDLAS) via direct absorption, yielding an actual OPL of 37.5 m with a deviation of only 2.1%. In the LITES configuration, this extended OPL translates into a stronger photothermal effect on the QTF surface, improving SNR and thus lowering the MDL. The cylindrical MPC achieves this long OPL while maintaining a compact volume, yielding a record-high OPL/V ratio of 37.4 cm^–2^, which serves as a key metric for absorption efficiency per unit system footprint. The specifications of other reported MPCs are presented in Table 1. Notably, the cylindrical MPC exhibits the highest OPL/V ratio, effectively enhancing absorption signals while maintaining the compact structure of the LITES sensor.

**Table 1:** Parameters comparison between different reported MPCs

MPC types	*γ*	OPL (m)	V (mL)	Total OPL/V (cm^–2^)
Seven rings pattern MPC^63^	215	26.4	281	9.4
Segmented circular MPC^64^	64	9.9	140	7
Four-circle pattern MPC^65^	272	37.85	273	13.85
Nine-circle pattern MPC^66^	235	32.7	281.7	11.6
Cylindrical MPC (this paper)	240	38.3	102.6	37.4

**Fig. 3:**
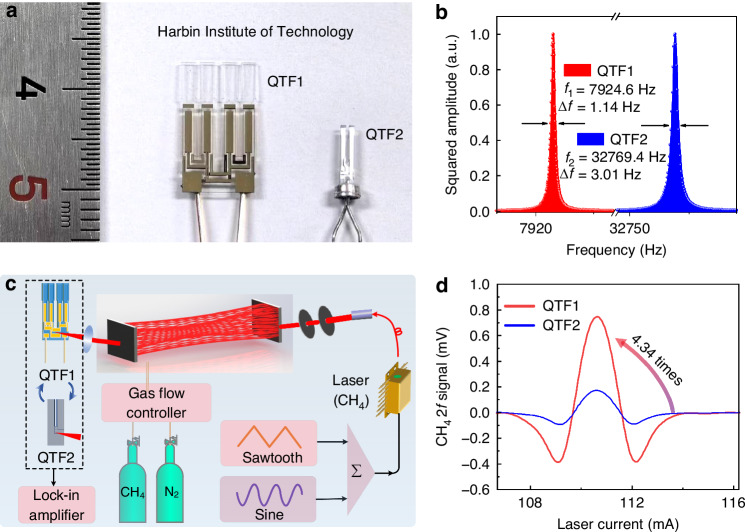
Characterization of the novel low-*f*_0_ four-tine QTF. **a** Photograph of the four-tine QTF. **b** Frequency responses of different QTFs. **c** Experimental setup for verifying the sensing performance of the four-tine QTF. **d** Comparison of signals from the different QTFs

To address the second challenge, a self-designed four-tine QTF was employed as the detector. Conventional LITES systems typically use commercial QTFs with *f*_0_ = ~32.768 kHz. However, its high *f*_0_ leads to short energy accumulation time and weak output signal. In this research, the *f*_0_ of the four-tine QTF is reduced through structural optimization. Additionally, the stress induced by prong vibration tends to concentrate in the narrow inner region where the tines connect to the support component. The four-tine QTF and the commercial QTF are designated as QTF1 and QTF2, respectively. As shown in Fig. 3a, in contrast to prior research, this study increases the number of QTF1’s tines. This design modification expands the contact area and enhances stress concentration, ultimately generating a larger piezoelectric charge and a stronger signal.

As illustrated in Fig. 3b, their experimentally measured *f*_0_ are 7924.6 Hz and 32769.4 Hz, respectively. Compared to the commercial standard QTF, the four-tine QTF exhibits a lower *f*_0_, which is conducive to energy accumulation. A schematic of the experimental setup used to evaluate the performance of the novel four-tine QTF is presented in Fig. 3c. Wavelength modulation spectroscopy (WMS) technology, a well-established method in absorption spectroscopy, was employed for this validation using CH_4_ as the target gas^53–56^. For a fair comparison, all parameters were kept identical except for the modulation frequencies, which were set to half the resonant frequency of each QTF for optimal excitation: 16384.7 Hz for the commercial QTF (*f*_0_ = 32769.4 Hz) and 3962.3 Hz for the four-tine QTF (*f*_0_ = 7924.6 Hz). The sawtooth scan frequency was maintained at 10 mHz, and the lock‑in amplifier integration time was set to 20 ms. The experimental results (Fig. 3d) reveal that the signal produced by the four-tine QTF is 4.34 times greater than that of the standard QTF. This confirms the superior conversion efficiency of the light-induced thermoelectric effect exhibited by the four-tine QTF.

Existing QTF enhancement strategies typically fall into three categories: geometry optimization^57–59^, surface absorption enhancement^60,61^ and alternative piezoelectric materials^62^. In contrast, this work introduces a fundamentally different approach by increasing the number of tines from two to four. This configuration enlarges the stress-concentration area at the prong base, generating a larger piezoelectric charge and stronger output signal. This four-tine design is orthogonal and complementary to existing strategies, offering potential for further performance gains through combination.

### CIPA design at the information layer

**Fig. 4:**
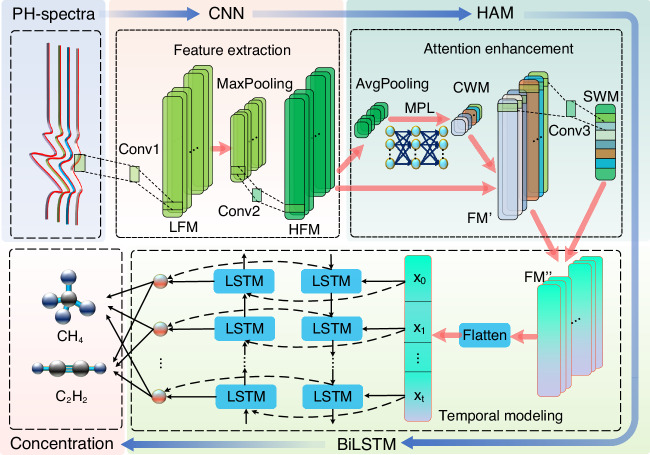
The information layer of the PH-LITES sensor

At the information layer, we present CIPA for high-precision, simultaneous inversion of CH_4_ and C_2_H_2_ gas concentrations in the PH-LITES sensor. As shown in Fig. 4, the core concept of this method is to establish an end-to-end nonlinear mapping from the raw parallel heterodyne spectra (PH-spectra) to gas concentration via framework of CNN-HAM-BiLSTM.

The feature extraction module employs a CNN structure: the first convolutional layer uses local receptive fields (with 3 × 1 kernels) and weight-sharing mechanisms to extract low-level feature maps (LFM) from the input spectra. A maximum pooling (MaxPooling) layer enhances translational invariance through down-sampling while preserving salient response regions and reducing parameter scale to prevent overfitting. The second convolutional layer further integrates multi-channel features to form high-level feature map (HFM), providing structured input for the subsequent attention mechanism.

At the feature optimization stage, a HAM is introduced to address the coupling of gas absorption features across channel and spatial dimensions. The channel attention module employs global average pooling (AvgPooling) to obtain global contextual representations of each feature map and uses multilayer perceptron (MLP) with bottleneck structures to obtain channel weights map (CWM). Channel attention weighted feature map (FM*ʹ*) can be expressed as:

5 $${\mathrm{FM}}^{\mathrm{'}}=\mathrm{HFM}\circ \mathrm{CWM}=\mathrm{HFM}\circ (\sigma (\mathrm{MLP}(\mathrm{AvgPooling}(\mathrm{HFM}))))$$

where *σ* denotes the sigmoid activation function, and ◦ represents the Hadamard product (element-wise multiplication). This mechanism essentially recalibrates the contribution of different feature channels to gas identification, grounded in frequency band selectivity and feature importance evaluation. The spatial attention module compresses the channel dimension via 1 × 1 convolution to generate a spatial weight map (SWM), which performs soft selection of feature significance along the wavelength direction. Spatial attention weighted feature map (FMʹʹ) can be expressed as:

6 $${\mathrm{FM}}^{{\prime\prime} }={{\rm{F}}}^{{\prime} }\circ \sigma (Conv(\mathrm{FM}^{\prime} ))$$

This enhances the model’s response to absorption peak regions and suppresses interference from non-sensitive bands. The two types of attention are fused multiplicatively, forming a dual-dimensional adaptive feature enhancement that effectively improves the model’s discriminative capability in mixed heterodyne spectra.

The temporal modeling module utilizes a BiLSTM network, based on the inherent temporal correlations in heterodyne spectra along the time sequence, which contain concentration-related information. BiLSTM employs gating mechanisms (forget, input, and output gates) to stably model long-range dependencies, avoiding gradient vanishing or explosion issues. The input gate is computed as:

7 $${i}_{t}=\sigma ({W}_{{i}}\cdot [{h}_{{\rm{t}}-1},{x}_{{t}}]+{b}_{i})$$

where *i*_*t*_ is the input gate at step *t*, *W*_*i*_ is the weight matrix, *h*_*t*−1_ is the hidden state at the previous time step, *x*_*t*_ is the input feature at step *t*, *b*_*i*_ is the bias term. The bidirectional structure aggregates spectral sequence information from both forward and backward directions. The final hidden state output contains global temporal information, providing discriminative features for concentration regression. The output layer maps high-dimensional features to the gas concentration space via fully connected transformation. The entire network is combined with the Adam optimizer and L₂ regularization to ensure generalization performance and convergence stability in complex spectral backgrounds. This improved deep learning-based concentration inversion algorithm enhances the capability for overlapping spectral features, offering a significant solution for fast multi-gas detection in the PH-LITES sensor.

### Performance of the PH-LITES sensor

In high-sensitivity gas detection technology based on LITES technology, the stability of the detector’s *f*_0_ plays a critical role. For the PH-LITES sensor, simultaneous and rapid detection of multi-component gases is achieved through the beat signal between the modulation frequency and the transient response of the QTF, with real-time monitoring of the QTF’s *f*_0_ also implemented. The modulation signals for the CH_4_ and C_2_H_2_ lasers are generated by superimposing the same varietal sawtooth wave and high-frequency sine wave. The varietal sawtooth wave has a period of 4 seconds, divided into two phases: the rapid rise phase enables lasers to quickly scan the absorption lines of CH_4_ and C_2_H_2_ simultaneously, generating a transient excitation signal for the QTF; and a stable phase, used to collect the QTF’s oscillation signal. The frequency of the sine wave (*f’*) is slightly offset from the QTF’s *f*_0_ by a small difference (Δ*f’*). The lock-in amplifier demodulates the transient signal generated at frequency *f’*, obtaining an exponentially decaying beat signal. The QTF used in the experiment is the high-performance four-tine QTF1 shown in Fig. 3, with the sine wave frequency *f’* set to 7926.3 Hz, corresponding to Δ*f’* = 1.7 Hz. It is worth noting that an excessively large Δ*f’* would shift the signal outside the QTF’s response bandwidth, weakening the signal, while an overly small Δ*f’* would extend the beat period and still lead to signal attenuation due to energy dissipation. The integration time of the lock-in amplifier is set to 5 ms, and the laser modulation depth is 4 mA. Conventional modulation methods face an inherent trade-off between signal amplitude and scanning speed due to the energy accumulation requirement of QTFs. The parallel heterodyne technique employs a varietal sawtooth wave with a rapid rise phase for fast scanning and a stable phase for signal collection, effectively decoupling this trade-off. While increasing the frequency of the varietal sawtooth wave could further accelerate scanning, this must be balanced against transient excitation efficiency and spectral resolution.

**Fig. 5:**
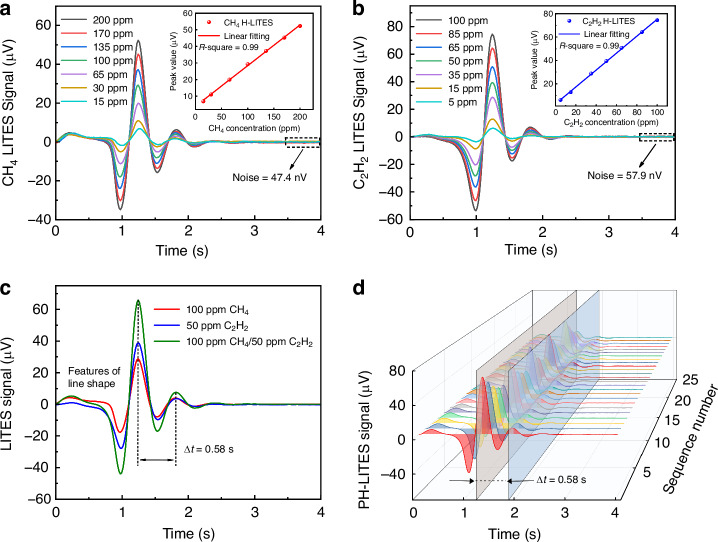
The comparison of different LITES signals. **a** CH_4_ heterodyne LITES signals of with different CH_4_ concentrations. **b** C_2_H_2_ heterodyne LITES signal with different C_2_H_2_ concentrations. **c** The comparison of LITES signals from mixed gas and single-gas samples. **d** Parallel heterodyne signals of CH₄/C₂H₂ mixtures

First, the heterodyne responses of the system to different single-component gases were tested, and the decay processes are shown in Fig. 5a, b. Linear fitting was performed between the first positive peak of the signal and the gas concentration, as depicted in the insets, yielding a coefficient of determination (R^2^) of 0.99. This confirms the high linear response characteristic of the system to single gases. The peak amplitudes for 100 ppm CH_4_ and 50 ppm C_2_H_2_ were 29.2 μV and 39.5 μV, respectively, with corresponding noise levels of 47.4 nV and 57.9 nV. The SNRs were calculated as 616 and 682, while the corresponding MDLs were determined to be 162.3 ppb and 73.3 ppb, respectively. This verifies the high detection sensitivity of the system’s physical layer, laying a high-quality data foundation for subsequent deep learning-based processing in the information layer. A comparison between the PH-LITES signal of the mixed gas and that of the single gas are shown in Fig. 5c. Since the beat signal period depends only on the intrinsic properties of the QTF and is independent of gas concentration, the decay peaks of different signals coincide in time. The time interval (Δ*t*) between the first and second peaks of the signal can be used to accurately determine the QTF’s *f*_0_. Here, Δ*t* is measured as 0.58 s. Based on the relationship Δ*t* = 1/Δ*f’*, the corresponding Δ*f’* is calculated as 1.7 Hz, matching the preset value. This proves that the *f*_0_ of the QTF in PH-LITES sensor can be detected by measuring the interval between the two peaks.

In addition, owing to differences in absorption line shape, line intensity, and other inherent spectral properties of different gases, the PH-LITES signal encapsulates distinguishable features that can be effectively extracted via deep learning for component identification. Parallel heterodyne signals of CH_4_/C_2_H_2_ mixtures with 25 different concentration ratios were tested, where the CH_4_ concentration ranges from 2 ppm to 50 ppm and the C_2_H_2_ concentration ranges from 5 ppm to 100 ppm. The resulting signal sequences are shown in Fig. 5d. All sequences exhibit the same Δ*t*, confirming the stability of the QTF’s resonant frequency *f*_0_. Ten repeated tests were performed for each concentration sequence, forming the training dataset for the information layer. This information layer, combined with the high-sensitivity signal acquisition capability of the physical layer, constitutes the complete PH-LITES sensor.

**Fig. 6:**
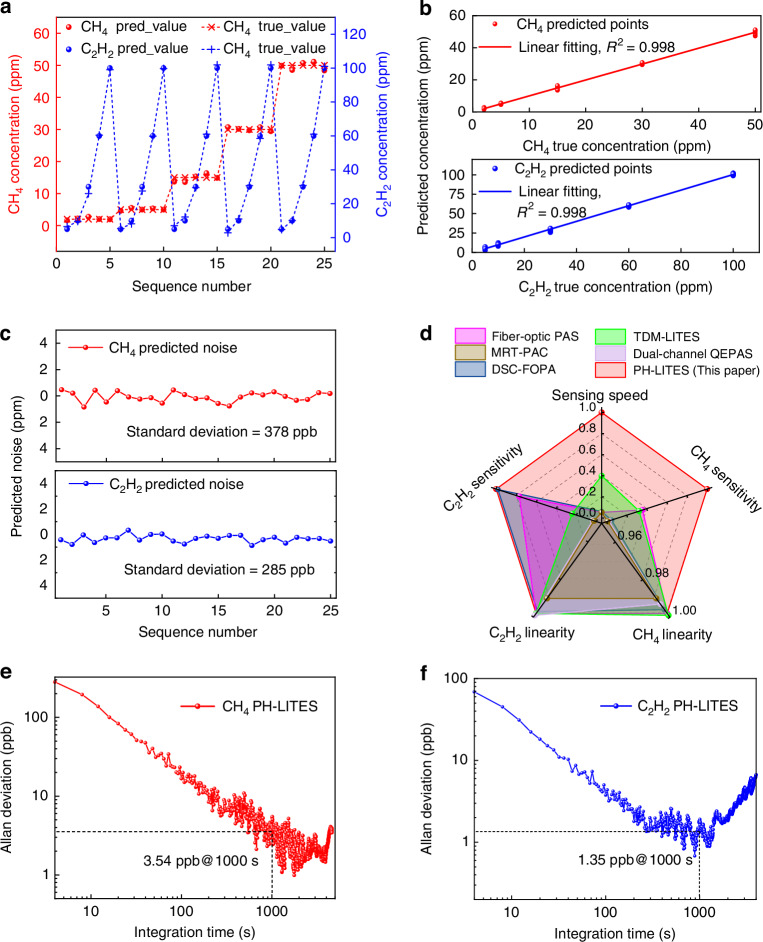
The performance of the PH-LITES sensor. **a** CH_4_ and C_2_H_2_ predicted concentration value. **b** The linear relationship between predicted and true concentration value. **c** Predicted noise level of the PH-LITES sensor. **d** Performance comparison among different spectroscopy techniques. **e** Allan deviation analysis of the predicted CH_4_ concentration. **f** Allan deviation analysis of the predicted C_2_H_2_ concentration

To verify the performance of the PH-LITES sensor, a series of validation experiments were carried out, and the relevant results are shown in Fig. 6. The sensor’s concentration prediction accuracy was tested across varying concentration ratios of CH_4_ and C_2_H_2_, and the results are presented in Fig. 6a. It can be seen from the figure that the PH-LITES sensor can accurately invert the concentrations of the two gases from the mixed transient response signal generated by QTF as shown in Fig. 5d. The mean relative errors (MRE) of the concentration prediction results for CH_4_ and C_2_H_2_ are as low as 8.9% and 5.8%. Figure 6b shows the correlation between predicted and actual concentration values. Linear fitting of the data yields a coefficient of determination (R^2^) of 0.998, further confirming the sensor’s excellent linear response to both gases. To validate the CNN‑HAM‑BiLSTM architecture, we compared it with several representative models using the same dataset. As shown in Table 2, the proposed model achieves the best performance across all metrics, demonstrating the effectiveness of the hybrid attention mechanism in enhancing task‑relevant features and suppressing noise in multi‑gas overlapping spectral scenarios.

**Table 2:** Comparison of different deep learning models for gas concentration prediction

types	MRE (CH_4_)	MRE (C_2_H_2_)	R^2^ (CH_4_)	R^2^ (C_2_H_2_)
CNN	11.0%	9.6%	0.995	0.997
BILSTM	9.3%	6.9%	0.996	0.998
CNN + BILSTM	10.9%	8.4%	0.997	0.996
CNN + HAM + BILSTM (This paper)	8.9%	5.8%	0.998	0.998

To clarify the detection limit of the sensor, its noise characteristics were tested under a pure nitrogen (N₂) background and shown in Fig. 6c. The standard deviations of the predicted concentration values for CH_4_ and C_2_H_2_ are 378 ppb and 285 ppb, respectively. These values correspond directly to the sensor’s MDLs in dual-gas simultaneous detection mode, providing key parameters for high-sensitivity applications. Notably, the MDLs reported in this work are calculated based on the 1σ criterion, which is consistent with the common practice in trace gas sensing, where MDL represents the concentration at which the signal is comparable to the noise level. It is worth noting that the detection sensitivity in dual-gas mode is slightly degraded compared to that in single-gas operation. This degradation primarily arises from algorithmic decoupling errors introduced by the CIPA model: dual-gas quantification requires the model to simultaneously decouple and reconstruct superimposed spectral features from a composite transient signal, which inevitably introduces minor reconstruction errors.

Figure 6d and Table 3 present a performance comparison between the PH-LITES sensor and other dual-gas sensors. The results indicate that achieving both rapid response and high detection sensitivity within a single sensor remains challenging. As shown, the PH-LITES sensor demonstrates significant advantages in both detection sensitivity and response speed. Furthermore, the sensor enables real-time monitoring of the detector’s resonant frequency *f*_0_, effectively calibrating the impact of potential frequency drift. The core innovation lies in identical-frequency excitation at the physical layer combined with algorithmic separation at the information layer, enabling simultaneous multi-gas detection within a single scan. Compared with time-division multiplexing, which requires *N* times the scan time, and frequency-division multiplexing, which needs multiple demodulation channels and only one gas at fundamental frequency, this method, combined with its high sensitivity, offers an efficient and robust solution for the rapid detection of low-concentration multi-component gases. To evaluate long-term stability, an Allan variance analysis was performed with pure N₂ over 6.5 hours. As shown in Fig. 6e, f, the MDLs for CH_4_ and C_2_H_2_ improve to 3.54 ppb and 1.35 ppb at an integration time of 1000 s. Notably, the use of deep learning-based concentration retrieval enhances robustness against non-concentration-related fluctuations, contributing to this extended optimal integration time.

**Table 3:** Performance comparison among different spectroscopy techniques for dual-gas detection

Sensor type	*f*_0_ drift calibration	Gas type	Integration time	Laser power	Scanning time	MDL	R^2^
Fiber-optic PAS^67^	None	CH_4_	1 s	9.4 mW	75 s	1.06 ppm	0.998
		C_2_H_2_		10.4 mW		0.37 ppm	0.998
MRT-PAC^68^	None	CH_4_	N/A	20 mW	75 s	40.79 ppm	0.99
		C_2_H_2_		30 mW		10.95 ppm	0.99
DSC-FOPA^69^	None	CH_4_	N/A	10.3 mW	65 s	7.64 ppm	0.996
		C_2_H_2_		200 mW		0.29 ppm	0.997
TDM-LITES^70^	None	CH_4_	N/A	N/A	10 s	1.17 ppm	0.999
		C_2_H_2_				1.17 ppm	0.998
Dual-channel QEPAS^71^	None	CH_4_	1 s	4 mW	200 s	17.47 ppm	0.993
		C_2_H_2_		4 mW		7.63 ppm	0.999
PH-LITES (This paper)	Yes	CH_4_	5 ms	16.2 mW	4 s	378 ppb	0.998
		C_2_H_2_		18.5 mW		285 ppb	0.998

ppm: 10^–6^, ppb: 10^–9^, N/A: Not available in the original literature

## Discussion

Beyond system integration, this work presents three fundamental contributions. First, it establishes synergistic co-design of physical and information layers: CSEA is deliberately engineered to provide raw signals with high SNR and rich information content via a cylindrical MPC with record-high OPL/V ratio (~37.4 cm^–2^) and a low-frequency four-tine QTF ( ~7.9 kHz), while the CIPA employs CNN-HAM-BiLSTM to maximally extract information from composite transient signals. Second, it demonstrates that a single QTF’s transient response suffices to decode multi-gas information—unlike conventional approaches that separate signals prior to detection, our approach allows signals to naturally superimpose and then employs intelligent algorithms to decouple information, proving that signal superposition is a feature to be exploited rather than a limitation. Third, it overcomes the inherent trade-off in gas sensing by simultaneously optimizing speed, species, and sensitivity: the PH-LITES sensor achieves a 4 s scan, ppb-level detection limits (378 ppb for CH_4_ and 285 ppb for C_2_H_2_), and excellent linearity (R² > 0.998) in a single compact system. Future work will extend this scheme to a larger number of gas species and incorporate k-fold cross-validation to enhance generalization and robustness. The PH-LITES architecture scales to more gases by adding lasers, with model complexity increasing accordingly. Model training data can be acquired during a one-time factory calibration, and long-term stability can be maintained through periodic multi-point checks using calibration gases. While further sensitivity improvement can be achieved by employing higher-reflectivity dielectric mirrors, shift the detection band to the mid-infrared region for stronger absorption, and integrating advanced denoising algorithms. By synergizing signal enhancement with intelligent processing, the PH-LITES sensor provides a novel and practical solution for applications requiring fast and sensitive multi-component gas analysis.

## Methods

### Development of the CSEA

The physical-layer sensitivity of the PH-LITES sensor is enabled by the CSEA, which integrates two key self-design components: a cylindrical MPC for absorption enhancement and a four-tine QTF for high-responsivity detection. The cylindrical MPC featured two orthogonally arranged cylindrical mirrors (M1: *f*_x_ = 360 mm; M2: *f*_y_ = 591 mm) with a precise separation of 159 mm. This configuration generated a dense, swirling laser spot pattern through astigmatism. The laser beam, coupled into the MPC via a 2-mm aperture on M1 at optimized incident angles of 2.18° relative to the x- and y-axes, underwent 240 reflections before exiting through an offset aperture on M2. This design achieved an extended OPL of 38.3 m within a compact internal V of 102.6 mL, yielding a record-high OPL/V of 37.4 cm^–2^. The silver-coated mirrors provided high reflectivity (~98%) across a broad wavelength range. The mirrors were fabricated via a high-precision optical cold working process, with their key surface figure accuracy indicators strictly controlled to a peak-to-valley (PV) value better than 3 λ and a surface irregularity (IRR) better than 0.5 λ. Alignment used a visible laser to match the simulated spot pattern before switching to detection lasers. The OPL of the cylindrical MPC was calibrated via direct absorption spectroscopy using CH_4_ (absorption line located at 1650.96 nm, with 200 ppm concentration). A near-infrared photodetector and a low-frequency sawtooth wave were used to scan the absorption line. Based on the Beer-Lambert law and line intensity from the HITRAN database, the calibrated OPL was 37.5 m with a deviation of 2.1%. Error sources include laser intensity fluctuations, gas temperature variations, and baseline fitting. This calibration does not affect the accuracy of subsequent indirect concentration measurements. The four-tine QTF was developed to address the limited thermoelastic conversion efficiency of standard dual-tine QTFs. The structural innovation involved the addition of two symmetric tines, which expanded the effective stress-concentration area during vibration. Each tine was designed with a widened T-shaped head to amplify the oscillation amplitude. Crucially, the tine length was increased to 10.9 mm (from 3.56 mm in a commercial QTF), which significantly reduced the *f*_0_ from ~32.8 kHz to ~7.9 kHz. This lower frequency extends the energy accumulation time, thereby enhancing the generated signal. The four-tine QTF was fabricated using standard photolithography and wet etching. A Z-cut quartz wafer was patterned via photolithographic exposure with a custom mask, followed by wet etching to form the tine structures (dimensional tolerance within ±3 μm). Gold electrodes were then deposited by physical vapor deposition to ensure conductivity and stability.

### Development of the CIPA

The CIPA constitutes the information layer of the PH-LITES sensor, engineered to decode the composite transient signals from the physical layer and perform accurate, simultaneous concentration retrieval for CH_4_ and C_2_H_2_. Its design follows an end-to-end “Feature extraction–Attention enhancement–Temporal modeling” pipeline, implemented via a hybrid CNN-HAM-BiLSTM network. The input to the CIPA is the 1D transient beat signal. The architecture begins with two convolutional blocks for hierarchical feature extraction. The CNN serves as the primary feature extractor. The first block employs 32 filters with a 3 × 1 kernel size, followed by ReLU activation and a 2×1 max-pooling layer. The second block uses 64 filters (3×1 kernel) to generate high-level feature maps. To address spectral overlap between gases, a dedicated HAM is inserted. The HAM sequentially channel attention and spatial attention to recalibrate feature importance across channels and along the spectral sequence. The refined features are then passed to a BiLSTM layer with 32 hidden units, which models the temporal dynamics of the signal decay. Finally, a fully-connected regression layer outputs the two concentration values. For each of the 25 concentration ratios, 10 independent measurement cycles were used for training and validation (9 for training, 1 for validation), and an additional 11th cycle was reserved as the independent test set for final performance evaluation. Beyond L₂ regularization, overfitting was mitigated through dimensionality reduction via pooling layers, a learning rate decay schedule, and validation against simpler baseline models (e.g., CNN-only and LSTM-only), which confirmed that the model learns meaningful features rather than memorizing noise. The current CIPA requires retraining for new gas combinations, which is acceptable for fixed-gas applications, while flexible scenarios can leverage a library of pre-trained models.

### Realization of the PH-LITES sensor

The sensor system employed two DFB lasers (laser 1 and 2), spectrally aligned to the absorption lines of CH_4_ and C_2_H_2_ by setting their operating temperatures and bias currents to 35 °C at 103 mA and 26 °C at 82 mA, respectively, yielding output powers of 16.2 mW and 18.5 mW. The absorption lines were selected based on the HITRAN database: for CH_4_, the selected line is located at 1650.96 nm (6057.08 cm^–1^) with a line strength of 1.5 × 10^–21 ^cm^–1^/(molecule·cm^–2^); for C_2_H_2_, the line is located at 1530.37 nm (6534.37 cm^–1^) with a line strength of 1.211 × 10^–20 ^cm^–1^/(molecule·cm^–2^). To address potential spectral interference, we examined absorption lines of common atmospheric gases (H_2_O, CO_2_, CO) in these regions and found no detectable absorption features near the selected CH_4_ and C_2_H_2_ lines, confirming no cross-interference between the two target gases nor interference from other species. The two beams were combined using a thin‑film filter MUX with an insertion loss of ~0.5 dB and co‑propagated through the cylindrical MPC. Modulation was implemented by superimposing a sine wave (7926.3 Hz, 4 mA amplitude) onto a variant sawtooth wave (0.25 Hz, 35 mA amplitude), which consists of equal 2-second rapid-rise and stable phases. The resulting transient photothermal signal from the QTF was demodulated using a lock‑in amplifier (Zurich Instruments MFLI) with a 5 ms integration time. For gas mixing, the concentrations of CH_4_ and C_2_H_2_ were independently controlled by adjusting the flow‑rate ratios of certified CH_4_/N_2_ and C_2_H_2_/N_2_ mixtures against a pure N₂ diluent stream, with each mixture line set to 300 sccm and a total flow of 600 sccm. All measurements were performed at atmospheric pressure (1 atm), and the QTF resonance frequency was characterized under the same pressure. Experiments were conducted at room temperature (25 °C) with a relative humidity of 16%.

## Data Availability

The data that support the findings of this study are available from the corresponding author upon reasonable request.
